# Responsibility and the limits of patient choice

**DOI:** 10.1111/bioe.12693

**Published:** 2019-11-26

**Authors:** Benjamin Davies

**Affiliations:** ^1^ Uehiro Centre for Practical Ethics University of Oxford Oxford United Kingdom of Great Britain and Northern Ireland

**Keywords:** choice, healthcare, public reasons, responsibility

## Abstract

Patients are generally assumed to have the right to choices about treatment, including the right to refuse treatment, which is constrained by considerations of cost‐effectiveness. Independently, many people support the idea that patients who are responsible for their ill health should incur penalties that non‐responsible patients do not face. Surprisingly, these two areas have not received much joint attention. This paper considers whether restricting the scope of responsibility to pre‐treatment decisions can be justified, or whether a demand to hold people responsible for 'usual suspect' choices such as smoking or failure to exercise commits us to also holding people responsible for their treatment choices. I argue that there is no good reason to support this restriction: those who advocate responsibility for (some) pre‐treatment choices should also advocate responsibility for (some) treatment choices. However, I also note that, as with pre‐treatment choices, patients may sometimes have reason to choose in ways that do not optimize their health. As such, I also consider a process, based on the idea of public reasons, for deciding which treatment choices patients cannot legitimately be held responsible for, along with a method for considering proposed changes to this category.

## 1 INTRODUCTION

In many healthcare systems, patients have a degree of choice over their treatment, including the option to refuse treatment altogether. This choice is subject to significant limits. It does not include treatment that is unnecessary and dangerous. In the U.K., body modification practitioner Brendan McCarthy (AKA ‘Dr Evil’) has recently been charged with grievous bodily harm for removing a client’s nipple, after the removal was refused by the NHS.1Cooper, J. (2019, Feb 13). Devon man who had nipple removed by 'Dr Evil' says he was 'really happy' with the work. *DevonLive*. Retrieved from https://www.devonlive.com/news/devon-news/devon-man-who-nipple-removed-2540712 [Accessed Apr 1, 2019]. Choice is often constrained by budget: patients cannot choose treatments that are insufficiently cost‐effective. Nonetheless, within many jurisdictions patients do have some choice over the form of their treatment.2Say, R. E., & Thompson, R. (2003). The importance of patient preferences in treatment decisions – challenges for doctors. *BMJ, 327*, 542–545; Coulter, A. (2010). Do patients want a choice and does it work? *BMJ, 341*, c4989; Poole, N. (2016). A common law right to autonomy of treatment. *Royal College of Surgeons Blog.* Retrieved from https://www.rcseng.ac.uk/news-and-events/blog/the-right-to-autonomy-of-treatment-is-a-common-law-right/; Radhakrishnan, A., Grande, D., Ross, M., Mitra, N., Bekelman, J., Stillson, C., & Pollack, C. E. (2017). ‘When primary care providers (PCPs) help patients choose prostate cancer treatment. *Journal of the American Board of Family Medicine, 30*, 298–307; Royal College of Surgeons. (2018). *Consent: Supported decision‐making. A guide to good practice.* London: Royal College of Surgeons. The UK’s National Health Service recently outlined plans to significantly reduce a number of ‘ineffective’ treatments whose risks typically outweighed benefits, but elected to keep four such treatments available upon patient request. See BBC. (2018, Jun 30). NHS England to stop ‘ineffective’ treatments’. Retrieved from https://www.bbc.co.uk/news/health-44665560. The NHS also offers patients with some conditions a degree of control over their treatment and care decisions with a Personal Health Budget (see https://www.nhs.uk/using-the-nhs/help-with-health-costs/what-is-a-personal-health-budget/). Where they do not, there is scope to offer patients more choice, either by permanently expanding the list of treatments that are standardly offered, or by giving particular patients one‐off extensions because of their circumstances or values.

The limit of cost‐containment raises a different issue. Some think that personal responsibility should play a role in healthcare allocation. According to proponents of this view, if you could have foreseen that your choices posed a risk to your health *and* made different, healthier choices, you should be held responsible for the outcome. Depending on further considerations, this might take the form of treatment denial, lower‐priority access, additional financial costs following treatment, or additional taxation of unhealthy but avoidable behaviours. Indeed, such thinking may influence the practice of *conditional access* to healthcare, where patients are denied the full package of available care until they have taken certain preventive measures such as losing weight or quitting smoking. Although such policies are often justified by reference to effectiveness, in some cases there may be latent assumptions of responsibility underlying these policies.3See Brown, R. (2019). Irresponsibly infertile? Obesity, efficiency and exclusion from treatment. *Health Care Analysis, 27*(2), 61–76. Moreover, some exclusionary policies are justified by reference to both patient obligations and costs to taxpayers.4Laverty, L., & Harris, R. (2018). Can conditional health policies be justified? A policy analysis of the new NHS dental contract reforms. *Social Science and Medicine, 207*, 46–54.


One justification for ‘responsibilisation’ is that those whose decisions generate avoidable health needs can *burden others*. One form of this argument points to social harm: your unhealthy choice avoidably increases the healthcare costs to society at large.5Knowles, J. (1977). The responsibility of the individual. *Daedalus, 106*(1), 57–80; Kluge, E. (1994). Drawing the ethical line between organ transplantation and lifestyle abuse. *Canadian Medical Association Journal, 150*(5), 745–746; Glannon, W. (1998). Responsibility, alcoholism, and liver transplantation. *Journal of Medicine and Philosophy, 23*(1), 31–49; Cappelen, A. W., & Norheim, O. F. (2005). Responsibility in health care: A liberal egalitarian approach. *Journal of Medical Ethics, 31*(8), 476–480; Buyx, A. M. (2008). Personal responsibility for health as a rationing criterion: Why we don’t like it and why maybe we should. *Journal of Medical Ethics, 34*(12), 871–874; Savulescu, J. (2018). Golden opportunity, reasonable risk and personal responsibility for health. *Journal of Medical Ethics, 44*(1), 59–61. For evidence on public support, see Ratcliffe, J. (2000). Public preferences for the allocation of donor liver grafts for transplantation. *Health Economics, 9*, 137–148; Berk, M., Gaylin, D., & Schur, C. (2006). Exploring the public’s views on the health care system: A national survey on the issues and options. *Health Affairs, 25*(1). https://doi.org/10.1377/hlthaff.25.w596; Traina, G., Martinussen, P., & Feiring, E. (2019). Being healthy, being sick, being responsible: Attitudes towards responsibility for health in a public healthcare system. *Public Health Ethics, 12*(2), 145–157. https://doi.org/10.1093/phe/phz009. See Wikler, D. (2002). Personal and social responsibility for health. *Ethics and International Affairs, 16*(2), 47–55 and Friesen, P. (2018). Personal responsibility within health policy: Unethical and ineffective. *Journal of Medical Ethics, 44*(1), 53–58 for critical discussions of the view. A second interpretation notes an individual harm: the treatment you require means that you take the place of a blameless individual, delaying or even obstructing their treatment.6Savulescu, J. (1998). The cost of refusing treatment and equality of outcome. *Journal of Medical Ethics, 24*, 231–236; Brudney, D. (2007). Are alcoholics less deserving of liver transplants. *Hastings Center Report, 37*(1), 41–47; Thornton, V. (2009). Who gets the liver? The use of responsibility as the tie breaker. *Journal of Medical Ethics, 35*, 739–742. The most obvious case is in the example of transplant waiting lists where, at the extreme, being lower down on the list can result in premature death. But this individualistic argument may apply quite generally: whatever their size, health and social‐care budgets are limited. If the health budget has little elasticity, resources spent treating avoidable health problems cannot be spent on unavoidable ones. If the health budget is elastic, but the broader social‐care budget is not, the same issue arises but with respect to goods outside the health service. Only with an infinitely (and, hence, unrealistically) elastic budget can we guarantee that treatment of those who are responsible for their health needs will not impact anyone with needs for which they are not responsible. In both cases, the thought is that giving equivalent healthcare to those who are responsible for their needs is an unjustified form of cost‐externalization.

Surprisingly, there has been little discussion of the combination of these issues, namely responsibility for treatment choices. Responsibility advocates have tended to focus on decisions made prior to entering the health system (‘pre‐clinical’ choices). Yet choosing a less efficient treatment or refusing an effective treatment seem to sometimes:

- be a genuine exercise of responsibility;
- generate additional, avoidable healthcare costs; and
- be amenable to guidance and support because patients have access to the expertise of medical professionals.

This paper considers whether this failure to extend the principles of responsibility to treatment choices is defensible, or mere myopia. I consider the plausibility of advocating that responsibility play a role in healthcare allocation for some pre‐clinical cases (smoking; excessive alcohol consumption; risk‐seeking behaviour; lack of exercise), while insisting that patients *not* be held responsible for choices regarding treatment. My conclusion is that this combination of views is not tenable. If we advocate that patients are held responsible for some pre‐clinical choices, we should also advocate that patients are held responsible for some treatment decisions. This is consistent with the view that ‘responsibilisation’ is justifiable in neither case, for example because we can never be sufficiently sure that someone is responsible for their condition, because the practical costs of finding out would negate any savings made, or because our health is insufficiently influenced by our individual choices.7For instance, one might point to research supporting the claim that our health is to a significant degree socially determined, e.g. Marmot, M. (2004). *Status syndrome: How your social standing directly affects your health and life expectancy*. London, U.K.: Bloomsbury; Benzeval, M., Bond, L., Campbell, M., Egan, M., Lorenc, T., Petticrew, M., & Popham, F. (2014). *How does money influence health?* York, U.K.: Joseph Rowntree Foundation. Still, while this evidence demonstrates that it is overly simplistic to regard people as individually responsible for their health (taken as a whole), that does not mean that people cannot be responsible for individual *choices* that affect their health, so long as those choices are made under conditions that are conducive to autonomous decision‐making. In other words, while the literature on social determinants of health shows us that we should not hold people responsible for failing to achieve *optimal* health, it does not show that we cannot hold (some) people responsible for failing to make specific choices that will likely *improve* their health.


Having established this claim, the paper then considers two further issues. In Section 3, I note that even if we can be responsible for our treatment choices, patients are often justified in choosing non‐optimal treatments. I therefore consider how we might go about deciding which treatment options patients can be held substantively responsible for in a pluralistic, liberal society, suggesting that the idea of public reason is the most promising route to take here. Finally, in Section 4, I consider how we ought to respond to patients whose treatment preferences are unreasonable. If our justification for excluding such treatments is that they will generate burdens that we should not reasonably have to bear, we can either allow people to pursue their preferred treatment, but insist that they shoulder the burdens themselves; or we can refuse to allow people their preferred, unreasonable treatment.

## 2 EXTENDING RESPONSIBILITY TO TREATMENT CHOICES

One might think that this problem has little practical importance. Healthcare systems generally set efficiency limits for fundable treatments, and doctors have standards of care that determine which treatments are acceptable. Any choice a patient has is within those limits. If the limits are defensible, patients should not be held responsible for choosing between options the healthcare system offers. If the limits are *not* defensible, it is not the fault of *patients* but of policymakers and/or medical professionals. Either way it is unfair to penalize patients.

However, this does not generally apply to the decision to *refuse* treatment altogether, even if this will lead to greater health needs in the future, for which the patient is entitled to care. There are exceptions to this – for instance, if a potential National Health Service (NHS) liver transplant candidate refuses help to stop drinking alcohol, they may be refused access to the transplant register8NHS Liver Advisory Group (2017). Liver transplantation: Selection criteria and recipient registration, Section 2.1.1.5. Retrieved from http://odt.nhs.uk/pdf/liver_selection_policy.pdf [Accessed Apr 1, 2019]. – but patients are not generally held responsible for past treatment refusals. It is worth noting in this context that a treatment refusal does not amount to a refusal to pursue *any* treatment, because some patients can pursue alternative treatments with private providers.9In fact, it is only recently (2017) that the National Health Service in England and Wales stopped funding homeopathy. See https://www.nhs.uk/conditions/homeopathy/, and it still funds acupuncture (https://www.nhs.uk/conditions/acupuncture/). The French government has announced its intention to stop reimbursing patients for homeopathy, but will not do so until 2021. See https://www.reuters.com/article/us-france-health-homeopathy/france-will-end-healthcare-refunds-for-homeopathic-drugs-idUSKCN1U42B6.


Additionally, the range of available treatments for any particular condition could be broader or narrower than it is in any particular medical jurisdiction. When we have alighted on a range of efficient treatments, some patients will want treatments outside this range. Patients may have *moral or religious objections* to treatments (e.g. objections to the use of animal products; Jehovah’s Witnesses’ objections to blood transfusions); they may have *different opinions* (informed or not) about the effectiveness of treatments or the risk of side‐effects (e.g. concerns about vaccinations); and where cost‐effectiveness is estimated at the population level, patients may have unusual *personal circumstances or preferences* that make a different treatment overall preferable. For instance, where treatments are judged less effective when they risk giving patients certain disabilities, some patients may be less concerned about developing a disability,10Davies, B. (2019). Bursting bubbles? QALYs and discrimination. *Utilitas, 31*, 191–202. leading them to consider treatments that are deemed inefficient.

In all such cases, we either already accept patients choosing significantly less efficient treatment options, or we could do so. And in all cases, we must decide whether the costs of those choices – if allowed – should fall on the patient or on others. In addition, it is worth considering the reasons for which doctors should offer a choice over treatment. Should doctors prioritize the treatment with the best expected outcome, offering patients a choice only when they are themselves uncertain about what is best? Or should patients be standardly offered a range of ‘good enough’ treatments, even when medical opinion suggests that one is clearly preferable?11Elwyn, G., Edwards, A., Gwyn, R. & Grol, R. (1999). Towards a feasible model for shared decision making: Focus group study with general practice registrars. *BMJ, 319*, 753–756.


Certain limits on patient choice are obviously objectionable. Take the view that patients have no right to refuse treatment at all: they must simply do what is best for their health, or accept it being done to them. This would involve violations of the basic right to bodily integrity, because ignoring a patient’s wish not to undergo a treatment would involve forcing it upon them. However, it is still possible to advocate more moderate versions of this view. Even though treatment cannot be physically forced on patients, they may nonetheless be refused ‘sub‐optimal’ treatments. For instance, Nair et al. describe a case in which a child’s parents are unhappy with a treatment that contains pork‐derived ingredients, where a bovine‐derived replacement is available but less effective.12Nair, T., Savulescu, J., Everett, J., Tonkens, R., & Wilkinson, D. (2017). Settling for second best: When should doctors agree to parental demands for suboptimal medical treatment? *Journal of Medical Ethics, 43*(12), 831–840. Such a case could clearly also occur for a patient making decisions about their own treatment, rather than about someone else’s. Similarly, Savulescu considers various alternatives to blood transfusions proposed by Jehovah’s Witnesses and concludes that it may be justifiable to reject claims to these alternatives if they force others to forego care.13Savulescu, op. cit., note 6. In both cases, we can rule out forcing patients to undergo optimal treatment but insist that their choice in the public health system is between this treatment and no treatment at all.

Because both pre‐clinical and treatment choices can foreseeably generate avoidable burdens, there is a *prima facie* case for treating them equivalently with respect to responsibility. This section considers three possible disanalogies between the two types of choice which might support the claim that, while we can hold people responsible for pre‐clinical choices, we cannot do the same for treatment choices.

### 2.1 Treatment choices are self‐regarding

One possible defence of the distinction between pre‐clinical behaviours and treatment choices is to say that the latter are ‘self‐regarding’.14Mill, J. S. (1859/2003). On liberty. In M. Warnock (Ed.), *Utilitarianism and on liberty* (pp. 88–180). Oxford, U.K.: Blackwell, p. 152. See also Cowart, D., & Burt, R. (1998). Confronting death: Who chooses? Who controls?’ *Hastings Center Report, 28*(1), 14–24. Whether I opt for the treatment my doctor recommends is, ultimately, my choice (though perhaps I have some obligation to consider the impact on my nearest and dearest). Yet just as apparently self‐regarding choices outside of clinical settings may end up having an impact on others if medical treatment is required, so too can refusals within clinical settings. A treatment refusal may impact others if it leads to greater health needs, *and* if the patient makes a claim for those new needs to be met. Choosing sub‐optimal treatment (including no treatment at all) can lead to worse health.

### 2.2 No clearly best treatment

A second possible difference between everyday choices and choices of medical treatment might be that we do not usually have sufficient knowledge to say which treatment is the best course of action.15A. Caplan cited in Glannon, op. cit., note 5, p. 42. The case for holding patients responsible for their treatment choices requires that the *outcomes* of those choices are reasonably foreseeable, for example through prognosis. This in turn presupposes that we can be sufficiently confident in prognosis to warrant penalty for failure to follow medical advice: if medical advice is not sufficiently well‐evidenced, then while it may nonetheless be prudent to follow such advice (assuming it is better than random guesswork) it may not be legitimate to penalize failure to do so.

Those who want to defend a distinction between the two types of choice might claim that we lack sufficient knowledge of the effects of medical care to justify the claim that refusing treatment *t* has sufficient risk of causing a particular health burden; but we *do* have the equivalent knowledge of at least some behaviours. For instance, we know that smoking daily has a very considerable risk of causing lung cancer.

If this were true, it would be an important practical objection to the claim that things are equivalent with respect to responsibility for treatment choice and other kinds of behaviour, though this would be an objection that is highly contingent on our current state of knowledge. However, this is not an essential difference between choices about treatment and other behaviours. For instance, while we may have good evidence about the causal relationship between smoking and cancer, things are far less clear with respect to relationships between other ‘usual suspect’ behaviours and apparently related health conditions, such as the link between diet and obesity.16Wilding, J. (2012). Are the causes of obesity primarily environmental? Yes. *BMJ*, *345,* e5843; Frayling, T. (2012). Are the causes of obesity primarily environmental? No. *BMJ*, *345*, e5843.


In addition, as I outline in Section 3, there is a difference between requiring that people always make the *best* choice – an unreasonable demand if even medical professionals disagree on what that choice is – and demanding that they make only *reasonable* choices. The latter is consistent with some uncertainty in prognosis.

### 2.3 Vulnerability

One objection to the practice of holding patients responsible even for pre‐clinical choices is that people are generally not in the right conditions to exercise sufficient responsibility for their health‐affecting choices. A possible response to this17Savulescu, op. cit., note 5. is to hold people substantively responsible only in carefully controlled conditions, for example when they have had their options carefully explained to them, when they are in a reasonable mental state to make an informed choice, and when we can rule out their having good reasons to avoid making the best choice for their health. This raises the possibility of a second objection to my claim, namely that such a mechanism is not possible for treatment decisions.

In one sense, decisions about treatment are better placed to meet the requirements of responsible choice. After all, such decisions take place partially in a clinical setting, where medical professionals are already on hand to explain the options to patients. Behaviours that are typically leapt on by advocates of responsibility, such as smoking, drug use, and poor diet,18Friesen, P. (2018). Personal responsibility within health policy: Unethical and ineffective’ *Journal of Medical Ethics, 44*, 53–58. result from decisions that are *not* typically made with such advice available, or in such a controlled setting. In this respect, then, treatment decisions seem to be a *more* appropriate subject of responsibility than standard behavioural decisions. Of course, this also emphasizes that if we are to hold people responsible for their treatment decisions, it must be clear not only what the medical risks are, but also what the institutional risks are. For example, if we pursue a policy of giving patients lower priority for conditions for which they have already refused treatment, that must be made clear to the patient at the time of choice.

But one might object that treatment decisions are often made at times of heightened vulnerability and sensitivity. The decision about which cancer treatment to pursue, for instance, is a fraught one, wrapped up in the emotional baggage of having the disease, of worrying about the effect on one’s family and friends, on work, and on one’s identity. It may seem unduly harsh to penalize people for making unwise choices in such circumstances.

However, my claim in this section is simply that there is an *at least equivalent* case for holding people responsible for *some* treatment decisions, compared with holding them responsible for non‐clinical choices. I thus make two observations about this concern. First, pre‐clinical choices are also often driven by emotions such as stress, unhappiness and fear. It is difficult to argue that this is *never* equivalent to the emotional difficulties caused by facing a disease.

Secondly, assume that some conditions are so emotionally difficult that patients cannot be regarded as sufficiently responsible when making treatment choices. This provides a case not for refusing to regard treatment decisions as equivalent to non‐treatment decisions with respect to responsibility, but for creating *exceptions* to a policy of holding patients responsible, or for establishing a *threshold* of emotional pressure above which patients cannot be held substantively responsible. We typically assume that responsibility requires a degree of *control.* 19Fischer, J. M., & Ravizza, M. (1998). *Responsibility and control: A theory of moral responsibility*. Cambridge, U.K.: Cambridge University Press. To the extent that emotional pressures make it difficult to choose well, this undermines the control condition on responsibility.

## 3 THE LIMITS OF PATIENT CHOICE

Even if you should be held responsible for health burdens that emanate from your choices, we must also accept that you can sometimes be *justified* in making choices that (foreseeably and avoidably) harm your health. Health is not the only good thing in life; you can reasonably trade off losses in health against other gains. And your life is not the only one that matters; you can reasonably trade off losses in your health against gains to others. In some such cases, we cannot reasonably insist that patients be penalized for making reasonable choices (particularly where those choices are forced on them by others or by circumstance).

This is no less true when it comes to decisions about treatment. A treatment that maximizes health may lead to other costs that are unacceptable to a reasonable patient. To rule out patient choice entirely would imply that patients have an obligation to maximize (expected) health whenever not doing so would lead to greater treatment. But such an obligation is hard to justify.

However, this does not mean that patients have an unlimited claim to any treatment they desire. Even if there is no obligation to optimize health, we should not impose unreasonable costs on others. This section considers how we might draw this distinction. If this distinction can be drawn in a non‐arbitrary way, we then face a choice with respect to those who seek to make medical decisions that carry unreasonable costs. Either we can seek to avoid imposing responsibility *and* to avoid incurring such costs by preventing people from carrying out such unreasonable decisions. Or we can take a responsibility‐friendly approach, allowing people to choose as they wish, but insisting that any costs incurred must fall on them. I consider these positions in Section 4.

Clearly, patients can refuse treatment that is *medically* optimal for good reasons. Health is not the only thing of value, and reasonable people can disagree about whether a particular trade‐off between goods is worthwhile. Moreover, people can reasonably prefer a worse life in order to improve the lives of others. Many liberals20Rawls, J. (1971). *A theory of justice.* Cambridge, MA: Harvard University Press. propose that the state ought to remain neutral in these sorts of cases, and not disadvantage people on the basis of their idiosyncratic conceptions of the good. At least on a straightforward reading, that prohibition might seem to cover penalizing patients for their idiosyncratic medical preferences.

Still, liberals typically include the caveat that the tolerated views must be ‘reasonable’.21An anonymous reviewer notes that a single moral value or principle might lead a patient to ‘unreasonably’ refuse a particular treatment, but also to adopt a lifestyle that is healthier than average. Such a patient might therefore end up generating fewer healthcare costs than they would have without this value. This is an important point in the overall context of justifying applying penalties on the basis of patient choices; but it is nonetheless true that a particular choice can be unreasonable even if it emanates from overall reasonable values. This raises the possibility that, while we must accommodate patients who refuse treatment on the basis of reasonable values and preferences, patients who refuse treatments *unreasonably* need not be allowed to do so. In a system that standardly offers patients a particular range of treatment options, such a policy might require that (a) patients explain the reasoning behind a decision to refuse any of those options (preferring either a non‐standard option or no treatment at all); (b) we have justifiable standards by which to judge the reasonableness of this reasoning; and (c) we have a procedure by which to apply these standards that is visibly procedurally fair.22Daniels, N., & Sabin, J. (2002) *Setting limits fairly: Learning to share resources for health.* Oxford, U.K.: Oxford University Press.


I will focus on the second and third of these three requirements, but it is worth briefly addressing the first. Requiring that patients explain their reasoning when requesting non‐standard treatment risks adding an additional layer of bureaucracy to medical decision‐making. One way to reduce this burden would be to shift the burden of reasonableness from the patient’s *reasons* to the decision they are making. Although we may want patients to make decisions based on good reasons, if patients choose a reasonable option, it may be irrelevant whether they choose for good reasons.

However, because we are considering *extensions* of the standard care options, there is a danger to this approach. A focus on decision types (e.g. to receive a bovine‐based vaccination rather than a pork‐based one) means that individual cases set a universal precedent, expanding the range of standard treatment options for everyone, indefinitely. Sometimes, it is reasonable to grant exemptions to general rules for some individuals but not for others. And if our initial justification for the practice of allowing exemptions is that some exemptions are reasonable, we must also acknowledge that other exemptions – even relating to the same treatment option for the same condition – could be unreasonable. This is an issue of whether we want to see this process as one of granting *individual exemptions*, or one of *challenging the range of accepted treatments*. In practice, both characterizations may be appropriate in different cases; but the difference is significant enough that it warrants different approaches.

A further restriction on this proposal is the ability of patients to articulate their reasons. Even patients who have reasonable grounds may struggle to articulate those grounds in a way that will satisfy adjudicators.23Huijer, M., & van Leeuwen, E. (2000). Personal values and cancer treatment refusal. *Journal of Medical Ethics, 26*, 358‐362. This issue will be exacerbated in cases where the justification for refusal is simply a brute preference. For instance, a patient may have a fear of staying in hospital. Even if we – and the patient – recognize that the fear is phobic, and so in some sense unreasonable, the patient will still find an extended hospital stay distressing, and hence overall worse for her welfare than an otherwise less effective treatment. Whether such a justification counts as ‘reasonable’ is a complex question, and one on which intuitive responses can easily be led astray.

The issue of articulation is also exacerbated because many patients in this situation will be seriously unwell. Patients who are sufficiently competent to form reasonable preferences about treatment may nonetheless find it difficult to express those preferences coherently because they are upset by their ill heath, and by the prospect of their preferences being refused. Additionally, forcing patients to undergo a process by which they justify their preferences may cause unwarranted additional stress.

Turning to the standards of reasonableness, although many writers in the liberal tradition place a constraint of reasonableness on views between which the state must remain neutral, the conception of reasonableness typically used is a broad one, leaving considerable space for a wide, varied pluralism of values. For instance, Rawls offers the notion of a ‘reasonable comprehensive doctrine’,24Rawls, J. (2005). *Political liberalism: Expanded edition.* New York, NY: Columbia University Press, p. xvi. namely a complete picture of value that a person holds. For a doctrine to be reasonable, however, it is only required that it addresses central aspects of life ‘in a more or less coherent and consistent manner’; that it offers some practical sense of what is centrally valuable in life, and of how to resolve clashes between competing values; and that it is subject to *some* adjustment in the light of good reasons (where what constitutes a good reason is itself judged from within the particular system of values). Clearly, this understanding of reasonableness is largely formal rather than substantive; that is, it concerns how beliefs are reached and maintained rather than what they are. Rawls does offer some substantive constraint in the form of the idea of the ‘reasonable citizen’, who is willing to engage in fair terms of cooperation in society so long as others are as well. This may rule out some substantive views (e.g. deeply bigoted ones) but will leave a great many standing.25Rawls’ approach is not designed to apply to anything so specific as people’s preferences over healthcare decisions. The point of considering his approach is simply to note that if we define reasonableness, as Rawls does, in a largely formal and permissive way, we will not get very far in ruling out particular choices as unreasonable.


A more promising approach to reasonableness lies in the idea of public reason, also discussed by Rawls, amongst others. One ideal of public reason is that where people want to direct public policy (which includes policies concerning medical spending), their preferences must be justifiable by reasons that ‘each person can reasonably endorse in their capacity as a free and equal citizen’.26Quong, J. (2010). *Liberalism without perfection.* Oxford, U.K.: Oxford University Press, p. 256. Even if individuals have additional reasons – based in more comprehensive versions of the truth as they see it – for their preferences, they must be capable of being justified on the basis of reasons that anyone who is minimally motivated could accept as reasonable, even if they ultimately disagree.27As Quong notes, Rawls sees the idea of public reason as applying only to the formation of the basic structure of society. Quong, however, argues that ‘the idea of public reasons…should regulate all the political decisions in a liberal democratic society’ (Ibid., p. 258).


One advantage of this approach is that it allows us to translate, to some degree, the otherwise irresolvable problem of evaluative disagreement into an issue of reciprocity. In medicine, there is not always a single treatment option that is uncontroversially best; even doctors may disagree on which course is best, or even over whether a particular choice is reasonable. I may think that the idiosyncratic preferences of others are irrational, and that my own idiosyncratic preferences are as‐yet unrecognized pearls of wisdom. On this basis, I might prefer a medical system that responds to my preferences, but which does not require me to fund others’ preferences: for instance, I may be a Jehovah’s Witness who believes that it is entirely reasonable for me to refuse blood transfusions in favour of more expensive alternatives,28Savulescu, op. cit., note 6. but reject someone else’s preference – also borne of a sincerely held doctrine – for homeopathic rather than pharmaceutical treatment for their cancer.

Further, while each individual might prefer not to support the idiosyncratic preferences of others at additional cost, we can all recognize the benefits of having a space to pursue our preferred way of life, where having such a space includes the collective shouldering of reasonable costs incurred. As such, it is preferable to require that those who would wish not to accept additional costs as a result of someone’s decision demonstrate that it is *un*reasonable, rather than that each individual demonstrate the reasonability of their suboptimal treatment preferences.

Yet I must also recognize that others feel the same: they would rather run things according to their preferences, ignoring the parts of my set of values that they regard false. We can each recognize that there is no unassailable claim to epistemic authority. In order to avoid my preferences being ignored entirely, I must be prepared to submit them to the same test as others’ preferences. The idea of publicly available reasons is one such test. I cannot appeal to considerations which, while I regard them as obviously true, can be reasonably rejected by others. For instance, I cannot appeal to specific religious doctrines,29This does not mean, however, that cultural and religious differences are to be ignored entirely. As I suggest below, in some cases such a difference can be reasonably accommodated because the degree of difference in medical efficacy or cost is not significant. The result of an appeal to public reason might be a *pluralistic* model that responds to different values and beliefs; indeed, given the liberal aim of accommodating as many reasonable views of the good as possible, this seems likely. The question, in this context, is rather about offering reasons for the externalization of special, and avoidable, costs. nor to unproven empirical claims, to back up my view.

This approach does not require a significant level of substantive moral agreement amongst individuals. Rather, it requires that individuals cooperating in a single system justify proposed changes to that system in terms that everyone can in principle understand and reasonably endorse. That means that individuals cannot appeal for special exemptions; they must justify proposed changes on the basis of reasons that in principle apply to all. However, it does not mean that people cannot appeal to special *features* of their case; it is simply that these special features must do equal justifying work in all similar cases. For instance, if Jehovah’s Witnesses appeal to religious conscience to justify their access to more expensive alternatives to blood transfusion, this must be compatible with equivalent religious exemptions for other groups.

## 4 BURDENSOME RIGHTS

Andrew Williams describes how, when the costs of certain choices are being unjustifiably externalized (i.e. borne by those who did not make the choice), we face two options.30Williams, A. (2008). Liberty, equality and property. In J. Dryzek, B. Honig & A. Phillips (Eds.), *The Oxford handbook of political theory* (pp. 488–506)*.* Oxford, U.K.: Oxford University Press. The first is to reduce the amount of freedom that people have to make such choices. The second is to retain the freedom to choose but reduce the degree to which the costs of such choices are externalized. The policy described in the previous section involves a method to identify when externalized costs are unjustified. A complete proposal thus needs to decide how to treat claims that fail to pass the test of public reasons. Some patients who fail this test will accept the result. But others will not: they will want to continue with their preferred treatment option even though it has been rejected as unreasonable.

One option is to allow the patient to choose as they wish but insist that patients must bear the burden of their unreasonable choices. For instance, we might allow patients to fund the difference in cost of more expensive treatments themselves. In cases of blood transfusion, this is straightforward, albeit costly: there are alternative treatments, but they are much more expensive. In other cases, though, things are less clear. One way that refusing a treatment might lead to higher costs is by leading to additional health needs (much) later. If a patient refuses treatment at time *t*, additional complications may arise, and existing health problems may be exacerbated. In some such cases, costs will be higher, but there is a practical challenge in estimating precisely how much of the additional cost is down to the patient’s refusal. Presumably, we will have to rely on prognoses with and without treatment, though this will never be exact.

There are obviously further questions about how far this principle should extend. Should my decision to refuse a treatment affect my rights to further treatment for the same condition a decade later? What about a separate condition, which may have been influenced by the first? We may worry that this risks moving from reasonable cost‐containment to an effective lifetime’s punishment for one mistake. Nonetheless, these are also issues that affect more standard accounts of responsibility in healthcare. Behavioural decisions made now can have health effects decades from now. We could recognize parity between clinical and non‐clinical decisions while still maintaining, for example, that there is a statute of limitations on how far in the future responsibility may be held; that there is a floor of severity below which responsibility should not apply; and that ordinary conditions of choice are not sufficient for holding patients responsible in this way.

A second option is to say that once a treatment option has been ruled out, it is ruled out whether or not the patient is willing and able to fund it. This option may receive support from considering the matter from a less individualistic perspective. A policy of allowing patients to make unreasonable treatment choices but then insisting that they bear the costs does not only affect the individual patient: poor health has emotional, social and financial implications for those around the patient as well. If we simply refuse to allow people to make unreasonable treatment choices, for example by banning public providers from offering such treatments even out of the patient’s own pocket, and perhaps even banning private provision of such treatments, we reduce the risk of those around the patient, who are *not* responsible for the patient’s choices or preferences, suffering the fallout.

If a treatment option is positive (i.e. requires provision by some other individual), and essentially involves taking excessive risks, the state may rule it out not by placing any restrictions on the patient’s behaviour, but by placing restrictions on the behaviour of others. The McCarthy case is of this general type, though it does not even involve a kind of treatment, but a medical procedure for a non‐medical purpose. Nonetheless, this might also be applied to some treatments that may benefit a genuine medical problem, but which are overly risky. In this case, the burdens the patient must bear are health burdens generated by failure to treat a medical condition. However, this proposal faces clear limits, given the possibility of simply refusing treatment altogether. Some patients who cannot access their preferred treatment option will simply accept what is available. But others will prefer not to receive treatment rather than accept what is on offer, and their health will suffer as a result. If the patient’s preferred option is to be left alone, we cannot regulate such an option away because this would involve the violation of a patient’s basic right to bodily integrity.

Perhaps a better way to mitigate the effect on individuals other than the particular patient is to place limits on the types of penalty for which a patient’s choices can make them liable. For instance, we might say that even if a patient’s severe health needs can reasonably be traced back to their unreasonable treatment preferences, they should not be refused basic care, but may have to make a greater financial contribution (dependent on ability to pay) towards the cost of that care.

## 5 CONCLUSION

Proponents of introducing responsibility as a rationing criterion for healthcare tend to focus on decisions made outside of the clinical setting. I have argued that these ‘usual suspect’ choices are not special, and that the same case can be extended to decisions made *within* the clinical setting, specifically decisions about which treatment route to take, if any. While choice of treatment is typically seen (within limits) as being ultimately the patient’s private business, unreasonable treatment demands can have an impact on others equivalent to that seen in more standard cases of health‐affecting behaviours. While I have not endorsed the introduction of responsibility as a criterion for decisions about treatment (or indeed other kinds of choice), I have suggested that the idea of public reason may help us to regulate the types of treatment to which we are standardly entitled in a public health service, as opposed to types of treatment for which we need to make a special case and which, if that is unsuccessful, we can potentially be held responsible for choosing. Whether this proposal is ultimately practicable depends on further questions about the nature of responsibility, and our ability to detect it. But insofar as these questions affect responsibility as a criterion quite generally, they do not present a special obstacle to holding people responsible for some types of treatment decision.

## CONFLICT OF INTEREST

The author declares no conflict of interest.

